# Fear of contagion, emotional stress and coping strategies used by adults during the first wave of the COVID-19 pandemic in Nigeria

**DOI:** 10.1186/s12888-022-04360-w

**Published:** 2022-11-24

**Authors:** Morenike Oluwatoyin Folayan, Olanrewaju Ibigbami, Brandon Brown, Maha El Tantawi, Nourhan M. Aly, Roberto Ariel Abeldaño Zuñiga, Giuliana Florencia Abeldaño, Eshrat Ara, Passent Ellakany, Balgis Gaffar, Nuraldeen Maher Al-Khanati, Ifeoma Idigbe, Anthonia Omotola Ishabiyi, Mohammed Jafer, Abeedha Tu-Allah Khan, Zumama Khalid, Folake Barakat Lawal, Joanne Lusher, Ntombifuthi P. Nzimande, Bamidele Olubukola Popoola, Mir Faeq Ali Quadri, Mark Roque, Joseph Chukwudi Okeibunor, Annie Lu Nguyen

**Affiliations:** 1 Mental Health and Wellness Study Group, Ile-Ife, Nigeria; 2 Faculty of Dentistry, Obafemi Awolowo University, Ile-Ife, Nigeria; 3 Department of Mental Health, Obafemi Awolowo University, Ile-Ife, Nigeria; 4 Department of Social Medicine, Population and Public Health, Riverside School of Medicine, University of California, Riverside, CA USA; 5 Department of Pediatric Dentistry and Dental Public Health, Faculty of Dentistry, Alexandria University, Alexandria, 21527 Egypt; 6 Postgraduate Department, University of Sierra Sur, Oaxaca, Mexico; 7 School of Medicine, University of Sierra Sur, Oaxaca, Mexico; 8 Department of Psychology, Government College for Women, Moulana Azad Road Srinagar Kashmir (Jammu and Kashmir), Srinagar, 190001 India; 9 Substitutive Dental Sciences, College of Dentistry, Imam Abdulrahman Bin Faisal University, Dammam, Saudi Arabia; 10 Department of Preventive Dental Sciences, College of Dentistry, Imam Abdulrahman Bin Faisal University, Dammam, Saudi Arabia; 11 Department of Oral and Maxillofacial Surgery, Faculty of Dentistry, Syrian Private University, Damascus, Syria; 12 Clinical Sciences Department, Nigerian Institute of Medical Research, Lagos, Nigeria; 13 Centre for Rural Health, School of Nursing and Public Health, University of KwaZulu-Natal, Durban, 4001 South Africa; 14 Department of Preventive Dental Sciences, Jazan University, Jazan, Saudi Arabia; 15 Department of Health Promotion, Faculty of Health, Medicine and Life Sciences, Maastricht University, Maastricht, The Netherlands; 16 School of Biological Sciences, University of the Punjab, Quaid-I-Azam Campus, Lahore, 54590 Pakistan; 17 Department of Periodontology and Community Dentistry, University of Ibadan and University College Hospital, Ibadan, Nigeria; 18 Provost’s Group, Regent’s University London, London, UK; 19 Department of Economic and Human Geography, Faculty of Geosciences, University of Szeged, 6722 Szeged, Hungary; 20 Department of Child Oral Health, University of Ibadan, Ibadan, Nigeria; 21 Division of Dental Public Health, Department of Preventive Dental Sciences, Jazan Universities, Jazan, Saudi Arabia; 22 Maternity and Childhood Department, College of Nursing, Taibah University, Madinah, Kingdom of Saudi Arabia; 23 WHO Regional Office for Africa, Brazzaville, BP 06 Congo; 24 Department of Family Medicine, Keck School of Medicine, University of Southern California, Los Angeles, USA

**Keywords:** Emotional Stress, Mental health, Coping strategies, Pandemic

## Abstract

**Background:**

The COVID-19 pandemic has induced high levels of stress. The aim of the study was to assess the relationship between emotional stress (COVID-19 related fear, anger, frustration, and loneliness) and the use of coping strategies among adults in Nigeria during the COVID-19 pandemic.

**Methods:**

Data from adults aged 18 years and above were collected through an online survey from July to December 2020. The dependent variables were COVID-19 related fear (fear of infection and infecting others with COVID-19), anger, frustration, and loneliness. The independent variables were coping strategies (use of phones to communicate with family and others, video conferencing, indoor exercises, outdoor exercises, meditation/mindfulness practices, engaging in creative activities, learning a new skill, following media coverage related to COVID-19) and alcohol consumption. Five logistic regression models were developed to identify the factors associated with each dependent variables**.** All models were adjusted for sociodemographic variables (age, sex at birth, and the highest level of education).

**Results:**

Respondents who consumed alcohol, followed media coverage for COVID-19 related information, and who spoke with friends or family on the phone had higher odds of having fear of contracting COVID-19 or transmitting infection to others, and of feeling angry, frustrated, or lonely (*p* < 0.05). Respondents who exercised outdoors (AOR: 0.69) or learned a new skill (AOR: 0.79) had significantly lower odds of having fear of contracting COVID-19. Respondents who practiced meditation or mindfulness (AOR: 1.47) had significantly higher odds of feeling angry. Those who spoke with friends and family on the phone (AOR: 1.32) and exercised indoors (AOR: 1.23) had significantly higher odds of feeling frustrated. Those who did video conferencing (AOR: 1.41), exercised outdoors (AOR: 1.32) and engaged with creative activities (AOR: 1.25) had higher odds of feeling lonely.

**Conclusion:**

Despite the significant association between emotional stress and use of coping strategies among adults in Nigeria during the COVID-19 pandemic, it appears that coping strategies were used to ameliorate rather than prevent emotional stress. Learning new skills and exercising outdoors were used to ameliorate the fear of contracting COVID-19 in older respondents.

## Introduction

Emotional stress is a feeling of psychological strain or tension that can challenge the ability to adapt and cope with certain situations and experiences [1]. The COVID-19 pandemic has been a source of emotional stress, inducing a range of feelings from fear to frustration and loneliness [2]. In addition to fears related to falling ill or dying from COVID-19, fears related to economic adversity were heightened during the pandemic [3]. There is also anxiety associated with the demand to understand and develop new habits and preventive behaviours over a short amount of time; behaviours that ordinarily may take longer to fully incorporate into habitual practice. For example, refraining from touching one’s face was strongly urged as a COVID-19 preventive behaviour. Yet, we touch our faces about 23 times in an hour [4] and it takes 68–254 days to permanently adopt a new behaviour [5]. Communication about risks and the behaviour changes required to cope with the pandemic challenged personal sense of stability and evoked fear and uncertainty for many [6].

Feelings of anger, frustration, and irritability increased during the pandemic in response to stress [7]. Individuals who experienced significant financial difficulties perceived themselves to be at greater risk for COVID-19. Those who obtained information about COVID-19 from social media and people of younger age were more likely to experience anger [8]. Stress may result from having to adapt to a new way of life during the extended period of the pandemic. The abrupt transition from in-person to remote modalities of studies and work forced many people to adjust and learn new technological skills and grapple with new roles and responsibilities. The move to the virtual environment also created limited access to the communities, people, and places that would usually be a source of comfort, relief, or support. One negative coping response to new and sudden stresses may be to lash out in anger [9].

Social isolation causes loneliness, characterized by feelings of emptiness, being unwanted, and cut off from other human beings [10]. Loneliness is a negative emotional response to the discrepancy between desired and attained relationships and is just one aspect of the “behavioural epidemic.” The phenomenon is termed as such because of the high global prevalence of loneliness and emotional dysfunction [11–16]. The behavioural epidemic refers to a multitude of mental health disorders such as depression, anxiety, substance abuse, domestic abuse, and suicide [17–20]. It is directly associated with the global increase in prevalence of chronic diseases since many mental health conditions are comorbid with many health conditions [21, 22]. Specific to COVID-19, a link has been shown between COVID-19 pandemic related loneliness and alcohol abuse and dependency symptoms, and avoidance behaviour as negative coping strategies [23]. Being required to quarantine or socially isolate for lengthy periods may induce emotional stress caused by loneliness, anger, and frustration [23]. In general, prevalence of emotional stress is lower in communitarian societies like Nigeria, where there is a greater emphasis on interdependence, tight social networks, and strong family connections [24].

The theoretical framework applied in this study is psychological stress theory [25]. The theory hypothesizes that emotional stress results when the demands of a particular environment exceed an individual’s ability to cope and respond, taxing their sense of wellbeing [25]. This relational transaction between the individual and their environment is appraised through the lens of an individual’s expectations and the significance they place on a specific encounter. Thus, the quality, intensity, and duration of emotional stress will differ between individuals in the same demanding environment [26]. Adaptation to emotional stress occurs through primary or secondary adaptation processes. Primary adaptation involves gaining control over the situation, perhaps through obtaining information to gain mastery, to alleviate stress while secondary adaptation is aimed at fitting in and coping with the situations [26].

Applied to this study, we consider the environment to be the health and social context created by the COVID-19 pandemic. Adaptive responses to the COVID-19 environment took many forms. Social media can be a source of knowledge and information. It also offers a mechanism to stay socially connected [27], although the use of the social media as a substitute for physical connection during the pandemic has been associated with negative impacts [28]. Other coping strategies employed to alleviate emotional stress associated with COVID-19 can include the consumption of alcohol and use of other psychoactive substances [29]. Positive coping strategies can include meditation or mindfulness practices [30, 31], exercise [32, 33], creative activities [34], learning new skills [35], phones and video conferencing [36], and or following media coverage of pandemic [37]. The aim of the study was to determine the association between emotional stress (fear, anger, frustration, and loneliness) and the use of coping strategies among adults in Nigeria during the first wave of COVID-19 pandemic. We hypothesised that 1) positive coping strategies would be associated with lower odds of emotional stress and 2) the use of negative coping strategies would be associated with higher odds of experiencing emotional stress.

## Methods

The data for these analyses were extracted from a multi-country global survey. The global survey assessed information about mental health and wellness from a global convenience sample of adults aged 18 years and older, from July to December 2020 [38]. Data were collected using an online survey platform. Study participants for the global survey were recruited through respondent-driven sampling. Initial participants reached by 45 data collectors were asked to share the survey link with their contacts within their countries. The survey links were also posted on social media groups (Facebook, Twitter, and Instagram), network email lists, and WhatsApp groups. Ethical approval for the study was obtained from the Human Research Ethics Committee at the Institute of Public Health of the Obafemi Awolowo University Ile-Ife, Nigeria (HREC No: IPHOAU/12/1557).

The data collection tool was initially developed for a study targeting a specific population in the United States [39] and was consequently adapted and validated for global use [40]. The overall validation score for the instrument was 0.83. The questionnaire took an average of 11 min to complete and was administered in English. Study participants were asked to complete an anonymous, closed-ended questionnaire about their mental health and well-being during COVID-19. Data collected into sociodemographic information, alcohol consumption, and COVID-19 related experiences of emotional stress (COVID-19 related fear—fear of getting infected and fear of giving someone else COVID-19, anger, frustration and loneliness). Data from a subset of participants who indicated that they lived in Nigeria were extracted for this study.

### Dependent variables

#### COVID-19 related fear

Respondents were asked if they experienced COVID-19 related fear during the pandemic; the fear of getting COVID-19 infection and the fear of giving COVID-19 to someone else (yes/no). The question was adopted from the Multi-Center AIDS Cohort Study [41].

#### COVID-19 related anger, frustration, and loneliness

The respondents were also asked if they had experienced anger, frustration and loneliness in response to the pandemic. The possible responses were: “Yes” or “No”.

### Independent variables

#### Coping strategies

The respondents were asked, “*what are the things you have done to take care of your mental health during the pandemic?*” with available options including “use of phones to communicate with family and others”, “video-conferencing”, “indoor exercises”, “outdoor exercises”, “meditation or mindfulness practices”, “engaging in creative activities”, “learning a new skill”, and “following media coverage related to COVID-19”. Respondents could select as many options as applicable.

#### Consumption of alcohol

Respondents were asked if they had experienced any change in the use of alcohol during the pandemic. The response options include “increase”, “decrease”, no change” and “not applicable”. These responses were further coded as “alcohol use” when they reported an increase, decrease or no change in alcohol consumption; and “no alcohol use” when they checked that alcohol consumption was not applicable.

### Confounders

#### Sociodemographic variables

Data collected included age, sex at birth, and highest level of education attained (none, primary, secondary, college/university).

### Data analysis

We performed multiple best-practice procedures to ensure the quality of the data collected [40]. Each participant could only complete the questionnaire once through IP address restrictions, though they could edit their answers freely until they choose to submit. We removed responses that were completed under seven minutes (*n* = 77) which was the lower limit of the time needed to complete the survey and removed data from participants with incomplete data on the study variables (*n* = 125).

Data were analysed using SPSS Version 23.0 (IBM SPSS Statistics for Windows, Armonk, NY: IBM Corp). Chi squared test (and t-test for age) were used to assess the association between the dependent variables (emotional stress—fear of getting COVID-19, fear of giving COVID-19 to someone else, anger, frustration and loneliness), and the independent variables (mental health maintaining modalities) as well as covariates (age, sex, educational status). Five binary logistic regression models were developed to identify the associations between the independent variables and each of the five dependent variables. Adjusted odds ratios (AOR) and 95% confidence intervals (CI) were calculated. The model fitness was assessed using the Nagelkerke R^2^, the Hosmer Lemeshow goodness of fit test and the Omnibus test of model coefficients. Statistical significance was set at < 5%.

## Results

Complete data were available from 4,471 participants. The age of the respondents ranged from 18 to 85 years with a mean of 37.3 (SD = 11.6) years. There were 2,395 (52.9%) non-male participants, 3,615 (80.9%) had a college/university education and 1,034 (23.1%) consumed alcohol. To cope during the pandemic, 2,557 (57.2%) respondents used their phones to interact with family members and significant others in their lives; 1,868 (41.8%) engaged in video conferencing, 900 (20.1%) engaged in meditation or mindful practices, 1,554 (34.8%) exercised indoors, 469 (10.5%) exercised outdoors, 1,225 (27.4%) engaged in creative activities, 1,102 (24.6%) learned new skills, and 2,094 (46.8%) followed media coverage related to COVID-19.

As shown in Table 1, respondents with fear of contracting the COVID-19 virus were significantly older (*p* < 0.001) and had no or primary education (*p* < 0.001). They were also significantly more likely to report alcohol consumption (*p* < 0.001), use the phone to interact with family members and significant others (*p* < 0.001), engage in video conferencing (*p* < 0.001), exercise indoors (*p* < 0.001), and follow media coverage related to COVID-19 (*p* < 0.001).

**Table 1:** Factors associated with COVID-19 related emotional stress (Fear of contracting COVID-19, Fear of infecting someone else with COVID-19, Anger, frustration and loneliness among Nigerians (*N* = 4471)

Variables	Total n (%)	Emotional stress														
		**Fear of contracting COVID-19**			**Fear of infecting someone else with COVID-19**			**Anger**			**Frustration**			**Loneliness**		
		Yes *n* = 2265 **n (%)**	No *n* = 2206 **n (%)**	*p* value	Yes *n* = 505 **n (%)**	No *n* = 3966 **n (%)**	*p* value	Yes *n* = 277 **n (%)**	No *n* = 4194 **n (%)**	*p* value	Yes *n* = 1036 **n (%)**	No *n* = 3435 **n (%)**	*p* value	Yes *n* = 644 **n (%)**	No *n* = 3827 **n (%)**	*p* value
**Age**	37.3 (11.6)	38.0 (11.6)	36.5 (11.6)	< 0.001	36.1 (10.3)	37.5 (11.8)	0.017	33.6 (11.2)	37.2 (11.6)	< 0.001	33.6 (11.6)	38.4 (11.4)	< 0.001	32.9 (11.4)	38.0 (11.5)	< 0.001
**Sex**																
Male	2076 (46.4)	1075 (51.8)	1001 (48.2)	0.162	283 (13.6)	1793 (86.4)	< 0.001	119 (5.7)	1957 (94.3)	0.232	500 (24.1)	1576 (75.9)	0.178	297 (14.3)	1779 (85.7)	0.863
Not Male	2395 (52.9)	1190 (49.7)	1205 (50.3)		222 (9.3)	2173 (90.7)		158 (6.6)	2237 (93.4)		536 (22.4)	1859 (77.6)		347 (14.5)	2048 (85.5)	
**Educational status**																
No formal education	48 (1.1)	37 (77.1)	11 (22.9)	< 0.001	5 (10.4)	43 (89.6)	0.994	9 (18.8)	39 (81.2)	< 0.001	13 (27.1)	35 (72.9)	0.001	15 (31.2)	33 (68.8)	< 0.001
Primary	84 (1.9)	66 (78.6)	18 (21.4)		9 (10.7)	75 (89.3)		11 (13.1)	73 (86.9)		20 (23.8)	64 (76.2)		15 (17.9)	69 (82.1)	
Secondary	724 (16.2)	421 (58.1)	303 (41.9)		81 (11.2)	643 (88.8)		51 (7.0)	673 (93.0)		209 (28.9)	515 (71.1)		143 (19.8)	581 (80.2)	
College/University	3615 (80.9)	1741 (48.2)	1874 (51.8)		410 (11.3)	3205 (88.7)		206 (5.7)	3409 (206)		794 (22.0)	2821 (78.0)		471 (13.0)	3144 (87.0)	
**Alcohol consumption**																
No alcohol consumption	3437 (76.9)	1649 (48.0)	1788 (52.0)	< 0.001	339 (9.9)	3098 (90.1)	< 0.001	171 (5.0)	3266 (95.0)	< 0.001	740 (21.5)	2697 (78.5)	< 0.001	442 (12.9)	2995 (87.1)	< 0.001
Yes, alcohol consumption	1034 (23.1)	616 (59.6)	418 (40.4)		166 (16.1)	868 (83.9)		106 (10.3)	928 (89.7)		296 (28.6)	738 (71.4)		202 (19.5)	832 (80.5)	
**Phone**																
No	1914 (42.8)	774 (40.4)	1140 (59.6)	< 0.001	136 (7.1)	1778 (92.9)	< 0.001	109 (5.7)	1805 (94.3)	0.230	350 (18.3)	1564 (81.7)	< 0.001	238 (12.4)	1676 (87.6)	0.001
Yes	2557 (57.2)	1491 (58.3)	1066 (41.7)		369 (14.4)	2188 (85.6)		168 (6.6)	2389 (93.4)		686 (26.8)	1871 (73.2)		406 (15.9)	2151 (84.1)	
**Video conferencing**																
No	2603 (58.2)	1259 (48.4)	1344 (51.6)	< 0.001	248 (9.5)	2355 (90.5)	< 0.001	148 (5.7)	2455 (94.3)	0.095	509 (19.6)	2094 (80.4)	< 0.001	310 (11.9)	2293 (88.1)	< 0.001
Yes	1868 (41.8)	1006 (53.9)	862 (46.1)		257 (13.8)	16 1 (86.2)		129 (6.9)	1739 (93.1)		527 (28.2)	1341 (71.8)		334 (17.9	1534 (82.1)	
Meditation or mindfulness practices																
No	3571 (79.9)	1786 (50.0)	1785 (50.0)	0.085	390 (10.9)	3181 (89.1)	0.116	200 (5.6)	3371 (94.4)	0.001	787 (22 0)	2784 (78.0)	< 0.001	475 ( 3.3)	3096 (86 7)	< 0.001
Yes	900 (20.1)	479 (53.2)	421 (46.8)		115 (12.8)	785 (87.2)		77 (8.6)	823 (91.4)		249 (27.7)	651 (72.3)		169 (18.8)	731 (81.2)	
**Indoor exercises**																
No	2917 (65.2)	1418 (48.6)	1499 (51.4)	< 0.001	314 (10.8)	2603 (89.2)	0.125	174 (6.0)	2743 (94.0)	0.381	588 (20.2)	2329 (79.8)	< 0.001	365 (13.5)	2522 (86.5)	0.024
Yes	1554 (34.8)	847 (54.5)	707 (45.5)		191 (12.3)	1363 (87.7)		103 (6.6)	1451 (93.4)		448 (28.8)	1106 (71.2)		249 (16.0)	1305 (84.0)	
**Outdoor exercises**																
No	4002 (89.5)	2045 (51.1)	1957 (48.9)	0.086	434 (10.8)	3568 (89.2)	0.005	241 (6.0)	3761 (94.0)	0.160	890 (22.2)	3112 (77.8)	< 0.001	543 (13.6)	3459 (86.4)	< 0.001
Yes	469 (10.5)	220 (46.9)	249 (53.1)		71 (15.1)	398 (84.9)		36 (7.7)	433 (92.3)		146 (31.1)	323 (68.9)		101 (21.5)	368 (78.5)	
**Creative activities**																
No	3246 (72.6)	1627 (50.1)	1619 (49.9)	0.243	353 (10.9)	2893 (89.1)	0.149	183 (5.6)	3063 (94.4)	0.012	668 (20 6)	2578 (79 4)	< 0.001	412 ( 2.7)	2834 (87 3)	< 0.001
Yes	1225 (27.4)	638 (52.1)	587 (47.9)		152 (12.4)	1073 (87.6)		94 (7.7)	1131 (92.3)		368 (30.0)	857 (70.0)		232 (18.9)	993 (81.1)	
**Learning new skills**																
No	3369 (75.4)	1729 (51.3)	1640 (48.7)	0.122	367 (10.9)	3002 (89.1)	0.138	208 (6.2)	3161 (93.8)	0.917	704 (20.9)	2665 (79.1)	< 0.001	447 (13.3)	2922 (86.7)	< 0.001
Yes	1102 (24.6)	536 (48.6)	566 (51.4)		138 (12.5)	964 (87.5)		69 (6.3)	1033 (93.7)		332 (30.1)	770 (69.9)		197 (17.9)	905 (82.1)	
**Following media coverage related to COVID-19**																
No	2377 (53.2)	1111 (46.7)	1266 (53.3)	< 0.001	220 (9.3)	2157 (90.7)	< 0.001	118 (5.0)	2259 (95.0)	< 0.001	437 (18.4)	1940 (81.6)	< 0.001	296 (12.5)	2081 (87.5)	< 0.001
Yes	2094 (46.8)	1154 (55.1)	940 (44.9)		285 (13.6)	1809 (86.4)		159 (7.6)	1935 (92.4)		599 (28.6)	1495 (71.4)		348 (16.6)	1746 (83.4)	

Respondents who had fear of infecting others with the virus were significantly younger (*p* = 0.017) and more likely to be male (*p* < 0.001). They were also more likely to consume alcohol (p < 0.001), use the phone to interact with family members and significant others (*p* < 0.001), engage in video conferencing (*p* < 0.001), exercise outdoors (*p* = 0.005), and follow media coverage of COVID-19 (*p* < 0.001).

Respondents who were younger (*p* < 0.001), with no formal education (*p* < 0.001), consumed alcohol (*p* < 0.001), meditated and used mindful practices (*p* < 0.001), engaged with creative activities (*p* < 0.001), and followed media coverage of COVID-19 (*p* < 0.001) were more likely to report feeling anger, frustration, and loneliness during the pandemic.

Respondents who used the phone to interact with family members and significant others and engaged in video conferencing (*p* < 0.001), exercised indoors and outdoors, and learned new skills (*p* < 0.05) were more likely to report feeling frustrated and lonely during the pandemic.

Results from the logistic regression models indicate that the proportion of variance in the dependent variables explained by the independent variables were low. New models with the independent variables included are an improvement over the baseline model as indicated by the Nagelkerke R^2^, Lemeshow goodness of fit test, and the Omnibus test of model coefficients.

Table 2 shows that respondents who consumed alcohol and who followed media coverage for COVID-19 related information had greater odds for fear of contracting COVID-19 or transmitting infection to others, and had greater odds for feeling angry, frustrated, and lonely (*p* < 0.05). Increase in age (AOR; 1.01; 95% CI: 1.01–1.02; *p* < 0.001) was associated with increased odds for fear of contracting COVID-19 but lower odds for fear of transmitting COVID-19, feeling angry, frustrated, and lonely. College/ university educated participants had lower odds for fear of contracting COVID-19 (*p* < 0.001), transmitting COVID-19 (*p* = 0.972), feeling anger (*p* = 0.001), frustration (*p* = 0.044), and loneliness (*p* < 0.001).

**Table 2:** Logistic regression analysis showing the factors associated with COVID-19 related emotional stress (Fear of contracting COVID-19, Fear of infecting someone else with COVID-19, Anger, frustration and loneliness among Nigerians (*N* = 4471)

Variables	Fear of getting COVID-19		Fear of giving COVID-19 to someone else		Anger		Frustration		Loneliness	
	**AOR (95% CI)**	*p* value	**AOR (95% CI)**	*p* value	**AOR (95% CI)**	*p* value	**AOR (95% CI)**	*p* value	**AOR (95% CI)**	*p* value
**Age**	1.01 (1.01–1.02)	< 0.001	0.98 (0.97–0.99)	0.004	0.97 (0.96–0.98)	< 0.001	0.96 (0.95–0.97)	< 0.001	0.96 (0.95–0.97)	< 0.001
**Sex at birth**										
Male (ref: Not male)	1.04 (0.92–1.18)	0.537	1.50 (1.24–1.82)	< 0.001	0.78 (0.60–1.01)	0.055	1.11 (0.95–1.28)	0.190	0.98 (0.82–1.18)	0.850
**Educational status**										
Primary (ref: no formal education)	1.19 (0.49–2.85)	0.704	1.05 (0.32–3.43)	0.930	0.77 (0.28–2.11)	0.608	0.84 (0.36–1.96)	0.688	0.50 (0.21–1.19)	0.117
Secondary (ref: no formal education)	0.49 (0.24–1.01)	0.052	0.96 (0.36–2.57)	0.929	0.27 (0.12–0.62)	0.002	0.65 (0.33–1.31)	0.229	0.34 (0.17–0.68)	0.002
College/University (ref: no formal education)	0.28 (0.14–0.57)	< 0.001	0.98 (0.37–2.58)	0.972	0.25 (0.11–0.54)	0.001	0.50 (0.25–0.98)	0.044	0.24 (0.12–0.47)	< 0.001
**Alcohol consumption**										
Yes (ref: no alcohol consumption)	1.55 (1.34–1.80)	< 0.001	1.52 (1.23–1.87)	< 0.001	2.11 (1.62–2.76)	< 0.001	1.34 (1.3–1.59)	0.001	1.53 (1.26–1.85)	< 0.001
**Phone**										
Yes (ref: No phone use)	2.07 (1.80–2.39)	< 0.001	2.17 (1.72–2.74)	< 0.001	0.98 (0.74–1.31)	0.906	1.32 (1.11–1.56)	0.002	1.04 (0.85–1.27)	0.736
**Video conferencing**										
Yes (ref: No video conferencing)	1.01 (0.87–1.17)	0.904	1.17 (0.93–1.46)	0.176	1.06 (0.78–1.43)	0.720	1.16 (0.97–1.38)	0.100	1.41 (1.15–1.74)	0.001
Meditation or mindfulness practices										
Yes (ref: No)	0.99 (0.84–1.18)	0.963	0.97 (0.76–1.23)	0.777	1.47 (1.08–2.01)	0.016	0.93 (0.77–1.12)	0.423	1.18 (0.95–1.47)	0.138
**Indoor exercises**										
Yes (ref: No)	1.06 (0.92–1.24)	0.423	0.83 (0.66–1.03)	0.096	0.94 (0.69–1.51)	0.661	1.23 (1.04–1.46)	0.017	0.89 (0.73–1.10)	0.293
**Outdoor exercises**										
Yes (ref: No)	0.69 (0.56–0.85)	0.001	1.15 (0.86–1.54)	0.341	1.02 (0.69–1.51)	0.917	1.08 (0.86–1.36)	0.524	1.32 (1.01–1.71)	0.041
Creative activities										
Yes (ref: No)	0.93 (0.79–1.09)	0.353	0.89 (0.71–1.13)	0.339	1.21 (0.89–1.64)	0.235	1.15 (0.96–1.38)	0.122	1.25 (1.01–1.54)	0.042
Learning new skills										
Yes (ref: No)	0.79 (0.67–0.93)	0.004	0.92 (0.72–1.17)	0.485	0.73 (0.53–1.00)	0.053	1.10 (0.92–1.31)	0.312	0.98 (0.79–1.22)	0.882
Following media coverage related to COVID-19										
Yes (ref: No)	1.40 (1.22–1.60)	< 0.001	1.38 (1.12–1.69)	0.003	1.65 (1.25–2.17)	< 0.001	1.64 (1.40–1.93)	< 0.001	1.33 (1.10–1.61)	0.003
**Nagelkerke R**^**2**^	0.102		0.059		0.081		0.116		0.100	
**Hosmer Lemeshow goodness of fit test**	9.56	0.297	10.58	0.227	7.92	0.441	11.03	0.200	10.19	0.252
**Omnibus test of model coefficients**	354.11	< 0.001	134.61	< 0.001	137.33	< 0.001	355.51	< 0.001	259.13	< 0.001

*AOR* Adjusted odds ratio, *CI* Confidence interval

Respondents who spoke with friends or family on the phone (AOR: 2.07; 95% CI: 1.80–2.39; *p* < 0.001) had significantly higher odds for fear of contracting COVID-19. People who exercised outdoors (AOR: 0.69; 95% CI: 0.56–0.85; *p* = 0.001) and learned a new skill (AOR: 0.79; 95% CI: 0.67–0.93; *p* = 0.004) had significantly lower odds for fear of contracting COVID-19. Respondents who spoke with friends and family on the phone (AOR: 2.17; 95% CI: 1.72–2.74; *p* < 0.001) and males (AOR: 1.50; 95% CI: 1.24–1.82; p < 0.001) had significantly higher odds for fear of transmitting COVID-19 to someone else.

Respondents who practiced meditation or mindfulness (AOR: 1.47; 95% CI: 1.08–2.01; *p* = 0.016) had significantly higher odds of feeling angry. Those who spoke with friends and family on the phone (AOR: 1.32; 95% CI: 1.11–1.56; *p* = 0.002) and exercised indoors (AOR: 1.23; 95% CI: 1.04–1.46; *p* = 0.017) had significantly higher odds of feeling frustrated. Those who video conferenced (AOR: 1.41; 95% CI: 1.15–1.74; *p* = 0.001), exercised outdoors (AOR: 1.32; 95% CI: 1.01–1.71; *p* = 0.041), and engaged in creative activities (AOR: 1.25; 95% CI: 1.01–1.54; *p* = 0.42) had higher odds of feeling lonely.

## Discussion

Findings suggest that many respondents in the survey used positive and negative coping strategies in response to emotional stress rather than to prevent emotional stress. Respondents with COVID-19 related fears and those who reported feelings of anger, frustration, and/or loneliness had higher odds of consuming alcohol and actively seeking information about COVID-19 through the media. Respondents who had COVID-19 related fears and those who felt frustrated had higher odds of using phone communication while those who felt lonely had higher odds of using coping strategies that involved visual contact with other people like video communication and exercising outdoors. Those who felt anger had higher odds of using introspective coping strategy (meditation or mindfulness practices). The two strategies that were more closely associated with preventing emotional stress were exercising outdoors and learning new skills as they were associated with lower odds of having the fear of contracting COVID-19. Our study hypotheses were, therefore, partially supported.

The modest values of the Nagelkerke R^2^ suggest that there are other independent variables that are likely to be associated with emotional stress that were not included in the regression analysis, and this is one of the limitations of our study. Another limitation is the cross-sectional design. Like with all cross-sectional studies, we are unable to determine with certainty, the direction of the associations. The data were collected at a single point in time and thus, the relationships between these variables may have shifted as people adapt to the new realities – called the new normal – created by the pandemic. In addition, the non-probability sampling design limits the broad generalisability of study findings, as responses were not representative of the general population of Nigeria. Also, the use of web-based methods for recruitment and data collection may exclude participants with low socioeconomic status who may not have access to a smart phone or the internet [42]. The large sample size however, allows for a more precise estimate of effect so that findings are reasonably generalizable to the demographic represented in the study [43]. Despite the limitations, our findings suggests that a large proportion of people experienced emotional stress during the COVID-19 pandemic.

In this study, we were unable to identify the possible impacts of the use of coping strategies by those who felt emotionally stressed. However, prior research suggests that positive coping can decrease vulnerability to poor mental health outcomes [44] such as depression [45, 46] and other psychological disorders [47, 48]. Alcohol consumption is a method that some individuals use for distancing themselves from stressors or challenges [49]. It slows down the central nervous system, creating feelings of relaxation, but also reduces inhibition, judgment, and memory [50]. Relying heavily on alcohol consumption as a coping strategy is generally discouraged because of the risk for developing alcohol-related disorders. Alcohol consumption can become a maladaptive behaviour when an individual lacks alternative coping strategy [51]. It is recommended that as part of the COVID-19 response, the public should be informed of the use of positive adaptive coping strategies for the management of the emotional stress that may be faced during a pandemic.

A viable tool for disseminating information on coping strategies is the media. We found that more respondents dealing with emotional stress turned to the media to seek information about COVID-19. Information-seeking, coping behaviour during a crisis may reflect a spectrum of passive or reflexive monitoring of a situation to seek solutions to a specific problem [52]. This behaviour is time bound, and specific to cultural and education contexts [52, 53]. Most respondents did not seek COVID-19 related information from the media, and we do not know if they sought information through other sources. However, the educational variability in information-seeking behaviour as demonstrated in this study, and possibly the cultural variability also, makes it important to conduct context-specific studies to understand how people use information management to cope with the COVID-19 pandemic. This can help in identifying possible ways of disseminating factual COVID-19 related information during this infodemic period [54].

We observed a pattern whereby individuals who were afraid of contracting COVID-19 used coping strategies that reduced contact with humans (e.g., used the phone to interact with others, engaged in video conferencing, exercised indoors, and followed media coverage of COVID-19). This may be an indication that respondents who have a strong perception of risk for contract COVID-19 are possibly less likely to be risk-takers and they therefore adopt coping strategies that promote social distancing. We also acknowledge that not all forms of COVID-19 related fears are dysfunctional so future studies may need to make a distinction between functional and dysfunctional fears in the study analyses [55].

In the present study, exercising outdoors and learning new skills were associated with lower odds for fear of contracting COVID-19. Learning new skills can buffer the detrimental effects of stress through access to new information, knowledge, and skills to enhance feelings of competency, self-efficacy, and resilience [56]. There is little clarity on how outdoor exercise may ameliorate the fear of contracting COVID-19 though the bi-directional relationship between the variables may infer that individuals with less concern about contracting the virus, may be more comfortable with leaving their homes to exercises outside [57–60]. There are, however, other factors that may restrict people from outdoor activities such as the severity of lockdown implemented by the government [61]. This study finding needs to be explored further.

Like prior studies, we found a negative association between age and anger [62], frustration, loneliness [63] and fear [63, 64] and a positive association between age and fear of contracting COVID-19 [65]. Older individuals are less likely to have interpersonal estrangement that leads to anger [62] and there is a general decline in negative affect as a function of age [63, 66]. Thus, increased age is associated with less of a tendency to feel fearful, angry, or frustrated. It is not unusual that older age was associated with higher odds for fear of contracting COVID-19 as older adults are the more severely affected by the pandemic. Risk for mortality and morbidity related to COVID-19 was significant higher for older adults, especially in the earlier waves of the pandemic [67].

The influence of age, gender, and cultural background on loneliness should be acknowledged. Respondents from collectivist societies like Nigeria are less likely to feel lonely and among these societies, loneliness is less common in women than in men [68]. We found only that gender was associated with fear whereby men had higher odds of being fearful of giving COVID-19 to others. There are different social contexts that shape gender differences around fear [69]. It is possible that the COVID-19 context is one where men are more fearful than women of transmitting COVID-19 to their family and peers. In a patriarchal society like Nigeria, this may be feasible as men are typically the breadwinners [70] and are therefore, more likely to contract COVID-19 through physical and social interactions at the workplace. Further studies are needed to validate this postulation.

Finally, the study showed that respondents with higher education had lower odds of COVID-19 related emotional stress. This may be because they are able to access reliable information to manage situations that causes emotional stress. Some studies have suggested a relationship between higher education and greater control over feelings of anger [71]. Since anger is linked to frustration [72, 73], education may be a resource that reduces vulnerability to anger, frustration, and loneliness [74].

## Conclusion

Although we found a positive association between emotional stress (fear, anger, frustration, and loneliness) and the use of coping strategies among adults in Nigeria during the COVID-19 pandemic, it seems that coping strategies were used to ameliorate rather than prevent emotional stress. Two coping strategies may have been used to ameliorate fear of contracting COVID-19: learning new skills and exercising outdoors. Findings from the present study indicate that there may be a need to conduct further studies that can identify coping strategies to prevent COVID-19 related stress. Evidence-based information about most effective strategies for preventing COVID-19 related stress could then be shared with the public.

## Data Availability

The datasets generated and/or analysed during the current study are not publicly available due to ongoing analysis using the dataset but are available from the corresponding author on reasonable request.

## Abbreviations

AOR: Adjusted odds ratio
CI: Confidence interval
COVID-19: Corona virus disease 2019

## References

[1] Salleh MR (2008). Life event, stress and illness. Malays J Med Sci.

[2] Duan L, Zhu G (2020). Psychological interventions for people affected by the COVID-19 epidemic. Lancet Psychiatry.

[3] Yoon E. Harvard Business Review; 2020. Behavioral Trends That Will Reshape Our Post-covid World. Available at: https://hbr.org/2020/05/3-behavioral-trends-that-will-reshape-our-post-covid-world. Accessed: 16 August 2021.

[4] Kwok YLA, Gralton J, McLaws ML (2015). Face touching: a frequent habit that has implications for hand hygiene. Am J Infect Control.

[5] Lally P, Van Jaarsveld CH, Potts HW, Wardle J (2010). How are habits formed: Modelling habit formation in the real world. Eur J Soc Psychol Eur.

[6] Arora A, Jha AK, Alat P, Das SS (2020). Understanding coronaphobia Asian J Psychiatr.

[7] Sajda Q (2020). Outrage and anger in a global pandemic: flipping the script on healthcare. Inform Technol Dev.

[8] Smith LE, Duffy B, Moxham-Hall V, Strang L, Wessely S, Rubin GJ (2021). Anger and confrontation during the COVID-19 pandemic: a national cross-sectional survey in the UK. J R Soc Med.

[9] Westbrook TD. Why is COVID-19 making me so angry? Available at: https://wexnermedical.osu.edu/blog/why-so-angry-covid. Accessed: 16 Aug 2021.

[10] Lonely. Merriam-Webster. Accessed May 13, 2021. Available at: https://www.merriam-webster.com/dictionary/lonely. Accessed: 10 July 2021.

[11] Jeste DV, Lee EE, Cacioppo S (2020). Battling the Modern Behavioral Epidemic of Loneliness: Suggestions for Research and Interventions. JAMA Psychiat.

[12] Leigh-Hunt N, Bagguley D, Bash K, Turner V, Turnbull S, Valtorta N, Caan W (2017). An overview of systematic reviews on the public health consequences of social isolation and loneliness. Public Health.

[13] Barreto M, Victor C, Hammond C, Eccles A, Richins MT, Qualter P (2021). Loneliness around the world: Age, gender, and cultural differences in loneliness. Pers Individ Dif.

[14] Mannes ZL, Burrell LE, Bryant VE, Dunne EM, Hearn LE, Whitehead NE (2016). Loneliness and substance use: the influence of gender among HIV+ Black/African American adults 50+. AIDS Care.

[15] Wu T, Jia X, Shi H, Niu J, Yin X, Xie J, Wang X (2021). Prevalence of mental health problems during the COVID-19 pandemic: A systematic review and meta-analysis. J Affect Disord.

[16] Nochaiwong S, Ruengorn C, Thavorn K, Hutton B, Awiphan R, Phosuya C, Ruanta Y, Wongpakaran N, Wongpakaran T (2021). Global prevalence of mental health issues among the general population during the coronavirus disease-2019 pandemic: a systematic review and meta-analysis. Sci Rep.

[17] Benjamin A, Kuperman Y, Eren N, Rotkopf R, Amitai M, Rossman H, Shilo S, Meir T, Keshet A, Nuttman-Shwartz O, Segal E, Chen A (2021). Stress-related emotional and behavioural impact following the first COVID-19 outbreak peak. Mol Psychiatry..

[18] Kuiper JS, Zuidersma M, Oude Voshaar RC, Zuidema SU, van den Heuvel ER, Stolk RP, Smidt N (2015). Social relationships and risk of dementia: A systematic review and meta-analysis of longitudinal cohort studies. Ageing Res Rev.

[19] Fässberg MM, van Orden KA, Duberstein P, Erlangsen A, Lapierre S, Bodner E, Canetto SS, De Leo D, Szanto K, Waern M (2012). A systematic review of social factors and suicidal behavior in older adulthood. Int J Environ Res Public Health.

[20] Weeks DG, Michela JL, Peplau LA, Bragg ME (1980). Relation between loneliness and depression: A structural equation analysis. Journal of Personality and Social Psychology.

[21] Steptoe A, Shankar A, Demakakos P, Wardle J (2013). Social isolation, loneliness, and all-cause mortality in older men and women. Proc Natl Acad Sci USA.

[22] Xia N, Li H (2018). Loneliness, Social Isolation, and Cardiovascular Health. Antioxid Redox Signal.

[23] Hoffart A, Johnson SU, Ebrahimi OV (2021). The network of stress-related states and depression and anxiety symptoms during the COVID-19 lockdown. J Affect Disord.

[24] BBC, University of Manchester. The BBC Loneliness Experiment. Accessed April 30, 2021. Available at: https://www.seed.manchester.ac.uk/education/research/impact/bbc-loneliness-experiment/. Accessed: 10 July 2021.

[25] Lazarus RS, Folkman S. Cognitive Theories of Stress and the Issue of Circularity. In: Appley MH, Trumbull R, editors. Dynamics of Stress. The Plenum Series on Stress and Coping. Boston: Springer; 1986. 10.1007/978-1-4684-5122-1_4.

[26] Lazarus RS, Launier R, Pervin LA, Lewis M (1978). Stress-related transactions between person and environment. Perspectives in Interactional Psychology.

[27] Pittman M, Reich B (2016). Social media and loneliness: Why an Instagram picture may be worth more than a thousand Twitter words. Comput Hum Behav.

[28] Cauberghe V, Van Wesenbeeck I, De Jans S (2021). How adolescents use social media to cope with feelings of loneliness and anxiety during COVID-19 lockdown. Cyberpsychol Behav Soc Netw.

[29] Sinha R (2008). Chronic stress, drug use, and vulnerability to addiction. Ann NY Acad Sci.

[30] Jones DR, Lehman BJ, Noriega A, Dinnel DL (2019). The effects of a short-term mindfulness meditation intervention on coping flexibility. Anxiety Stress Coping.

[31] Ramasubramanian S (2017). Mindfulness, stress coping and everyday resilience among emerging youth in a university setting: a mixed methods approach. Int J Adolesc Youth.

[32] Garber MC (2017). Exercise as a Stress Coping Mechanism in a Pharmacy Student Population. Am J Pharm Educ.

[33] Azizi M (2011). Effects of doing physical exercises on stress-coping strategies and the intensity of the stress experienced by university students in Zabol, Southeastern Iran. Procedia Soc Behav Sci.

[34] Martin L, Oepen R, Bauer K (2018). Creative Arts Interventions for Stress Management and Prevention-A Systematic Review. Behav Sci (Basel).

[35] Hidalgo-Andrade P, Hermosa-Bosano C, Paz C (2021). Teachers’ Mental Health and Self-Reported Coping Strategies During the COVID-19 Pandemic in Ecuador: A Mixed-Methods Study. Psychol Res Behav Manag.

[36] Kim JI, Yun JY, Park H (2018). A Mobile Videoconference-Based Intervention on Stress Reduction and Resilience Enhancement in Employees: Randomized Controlled Trial. J Med Internet Res.

[37] Eden AL, Johnson BK, Reinecke L, Grady SM (2020). Media for Coping During COVID-19 Social Distancing: Stress, Anxiety, and Psychological Well-Being. Front Psychol.

[38] Nguyen AL, Brown B, Tantawi ME, Ndembi N, Okeibunor J, Mohammed A, Folayan MO (2021). Time to Scale-up Research Collaborations to Address the Global Impact of COVID-19 – A Commentary. Health Behavior and Policy Review.

[39] Nguyen AL, Christensen C, Taylor J, Brown B (2020). Leaning on Community-Based Participatory Research to Respond During COVID-19. AIDS Behav.

[40] El Tantawi M, Folayan MO, Nguyen AL, Aly NM, Ezechi O, Uzochukwu BSC, Alaba OA, Brown B (2022). Validation of a COVID-19 mental health and wellness survey questionnaire. BMC Public Health.

[41] MACS/WIHS Combined Cohort Study. Available at: https://statepi.jhsph.edu/mwccs/. Accessed 15 May 2021.

[42] Greiner Safi A, Reyes C, Jesch E, Steinhardt J, Niederdeppe J, Skurka C, Kalaji M, Scolere L, Byrne S (2019). Comparing in person and internet methods to recruit low-SES populations for tobacco control policy research. Soc Sci Med.

[43] Biau DJ, Kernéis S, Porcher R (2008). Statistics in brief: the importance of sample size in the planning and interpretation of medical research. Clin Orthop Relat Res.

[44] Turk T, Elhady MT, Rashed S, Abdelkhalek M, Nasef SA, Khallaf AM (2018). Quality of reporting web-based and non-web-based survey studies: What authors, reviewers and consumers should consider. PLoS ONE.

[45] Farzin RB, Asgharnejad FAA, Yekkeh YR, Habibi AM (2010). Comparison of copying strategies and personality styles in depressed and non-depressed students. International Journal of Behavioral Sciences.

[46] Skapinakis P, Bellos S, Oikonomou A, Dimitriadis G, Gkikas P, Perdikari E, Mavreas V (2020). Depression and Its Relationship with Coping Strategies and Illness Perceptions during the COVID-19 Lockdown in Greece: A Cross-Sectional Survey of the Population. Depress Res Treat.

[47] Sim K, Chan YH, Chong PN, Chua HC, Soon SW (2010). Psychosocial and coping responses within the community health care setting towards a national outbreak of an infectious disease. J Psychosom Res.

[48] Zhong W, Kim Y, Jehn M (2013). Modeling dynamics of an influenza pandemic with heterogeneous coping behaviors: case study of a 2009 H1N1 outbreak in Arizona. Comput Math Organ Theory.

[49] Blaine SK, Sinha R (2017). Alcohol, stress, and glucocorticoids: From risk to dependence and relapse in alcohol use disorders. Neuropharmacology.

[50] Metzger IW, Blevins C, Calhoun CD, Ritchwood TD, Gilmore AK, Stewart R, Bountress KE (2017). An examination of the impact of maladaptive coping on the association between stressor type and alcohol use in college. J Am Coll Health.

[51] Merrill JE, Thomas SE (2013). Interactions between adaptive coping and drinking to cope in predicting naturalistic drinking and drinking following a lab-based psychosocial stressor. Addict Behav.

[52] Waldron VR, Dervin B, Smith LC (1988). Sensemaking as a framework for knowledge acquisition. Artificial intelligence, expert systems and other applications: American Society for Information Science Mid-Winter Meeting.

[53] Savolainen R (2006). Time as a context of information seeking. Libr Inf Sci Res.

[54] Prandi L, Primiero G (2020). Effects of misinformation diffusion during a pandemic. Applied Network Science.

[55] Solymosi R, Jackson J, Pósch K, Yesberg JA, Bradford B, Kyprianides A (2021). Functional and dysfunctional fear of COVID-19: a classification scheme. Crime Sci.

[56] Zhang C, Mayer DM, Hwang E (2018). More is less: Learning but not relaxing buffers deviance under job stressors. J Appl Psychol.

[57] Petersen JA, Naish C, Ghoneim D, Cabaj JL, Doyle-Baker PK, McCormack GR (2021). Impact of the COVID-19 Pandemic on Physical Activity and Sedentary Behaviour: A Qualitative Study in a Canadian City. Int J Environ Res Public Health.

[58] Eder SJ, Steyrl D, Stefanczyk MM, Pieniak M, Martínez Molina J, Pešout O (2021). Predicting fear and perceived health during the COVID-19 pandemic using machine learning: A cross-national longitudinal study. PLoS ONE.

[59] Sallis R, Young DR, Tartof SY, Sallis JF, Sall J, Li Q, Smith GN, Cohen DA (2021). Physical inactivity is associated with a higher risk for severe COVID-19 outcomes: a study in 48 440 adult patients. Br J Sports Med..

[60] Dominski FH, Brandt R (2020). Do the benefits of exercise in indoor and outdoor environments during the COVID-19 pandemic outweigh the risks of infection?. Sport Sci Health..

[61] Rice WL, Mateer TJ, Reigner N, Newman P, Lawhon B, Taff BD. Changes in recreational behaviors of outdoor enthusiasts during the COVID-19 pandemic: analysis across urban and rural communities, Journal of Urban Ecology. 2020; 6(10): juaa020, 10.1093/jue/juaa020

[62] Schieman S (1999). Age and anger. J Health Soc Behav.

[63] Barreto M, Victor C, Hammond C, Eccles A, Richins MT, Qualter P (2021). Loneliness around the world: Age, gender, and cultural differences in loneliness. Personality Individ Differ.

[64] Hudson NW, Lucas RE, Donnellan MB (2016). Getting older, feeling less? A cross-sectional and longitudinal investigation of developmental patterns in experiential well-being. Psychol Aging.

[65] Charles ST, Reynolds CA (2001). Gatz Age-related differences and change in positive and negative affect over 23 years. M J Pers Soc Psychol.

[66] Lawton MP, Kleban MH, Dean J (1993). Affect and age: cross-sectional comparisons of Structure and prevalence. Psychol Aging.

[67] Kang SJ, Jung SI (2020). Age-related morbidity and mortality among patients with COVID-19. Infection & chemotherapy.

[68] Turk CL, Heimberg RG, Orsillo SM, Holt CS, Gitow A, Street LL, Schneier FR, Liebowitz MR (1998). An investigation of gender differences in social phobia. J Anxiety Disord.

[69] [No author]. Male breadwinner, female homemaker: Patriarchy and women’s work-life balance in Nigeria. Hum Resour Manag Int Dig. 2019;27(7):9–11.

[70] Eboiyehi FA, Muoghalu CO, Bankole AO (2016). In their husbands' shoes: Feminism and political economy of women breadwinners in Ile-Ife, Southwestern Nigeria. J Int Women's Stud.

[71] Boylan JM, Ryff CD (2013). Varieties of anger and the inverse link between education and inflammation: toward an integrative framework. Psychosom Med.

[72] Bertsch K, Back S, Flechsenhar A, Neukel C, Krauch M, Spieß K, Panizza A, Herpertz SC (2021). Don't Make Me Angry: Frustration-Induced Anger and Its Link to Aggression in Women With Borderline Personality Disorder. Front Psych.

[73] Blair RJR (2018). Traits of empathy and anger: implications for psychopathy and other disorders associated with aggression. Philos Trans R Soc Lond B Biol Sci.

[74] Bishop AJ, Martin P (2007). The indirect influence of educational attainment on loneliness among unmarried older adults. Educ Gerontol.

